# Low skeletal muscle density is a risk factor for constipation in patients on maintenance dialysis

**DOI:** 10.3389/fphys.2025.1725508

**Published:** 2025-12-05

**Authors:** Mengting Li, Qing Yin, Shimei Hou, Lirong Hao, Jianbing Hao, Liuping Zhang, Wei He, Qinglei Xie

**Affiliations:** 1 Institute of Nephrology, Zhong Da Hospital, Southeast University School of Medicine, Nanjing, Jiangsu, China; 2 Department of Nephrology, Southern University of Science and Technology Hospital, Shenzhen, China; 3 Department of Gastroenterology, Geriatric Hospital of Nanjing Medical University, Nanjing, Jiangsu, China

**Keywords:** constipation, hemodialysis, peritoneal dialysis, computed tomography, skeletal muscle

## Abstract

**Background:**

Constipation is among the most common gastrointestinal disorders among dialysis patients. Low skeletal muscle density (SMD), a marker of increased intramuscular fat infiltration in muscle, is also among the important characteristics of dialysis patients. This study aimed to assess whether a low SMD is associated with constipation in patients on maintenance dialysis.

**Methods:**

A total of 428 dialysis patients from three dialysis centers in our hospital were enrolled in this cross-sectional study. Constipation was assessed according to the Rome IV diagnostic criteria. The SMD was determined via computed tomography (CT) at the first lumbar vertebra level. Univariate and multivariate logistic regression analyses were used to explore the potential effect of SMD on constipation.

**Results:**

The mean age of all participants was 56.09 (±15.17) years. The percentage of male participants was 42.99%. In accordance with the Rome IV diagnostic criteria, the prevalence of constipation among dialysis patients was 62.62%. We found that compared with that of patients with no constipation, the SMD of patients with constipation was significantly lower [31.70 (±7.33) HU vs. 38.44 (±6.44) HU, P < 0.001]. Decreased SMD was significantly associated with constipation in dialysis patients. The association remained statistically significant even after adjusting for age, dialysis vintage, hemoglobin concentration, serum phosphate concentration and history of diabetes, skeletal muscle index and body mass index. The odds ratios were 0.26 (0.10–0.70), 0.25 (0.08–0.80) and 0.21 (0.05–0.80) for SMD quartile 2, quartile 3 and quartile 4, respectively (reference, quartile 1). Furthermore, the area under the curve (AUC) of the nomogram in the training group was 0.74, whereas that in the test group was 0.76.

**Conclusion:**

Dialysis patients have a high prevalence of constipation. We found that a low SMD is an independent risk factor for constipation. Our findings provide a new perspective on the causes of susceptibility to constipation among dialysis patients.

## Introduction

Constipation, a functional bowel disorder characterized by difficult or reduced bowel movements, is among the most common gastrointestinal disorders among dialysis patients (Cha et al., 2023; Sumida et al., 2019a). Increasing evidence has indicated that the prevalence of constipation in patients with chronic kidney disease (CKD) is greater than that in the general population. Specifically, reports have shown that the prevalence of constipation ranges from 1.6% to 71.7% in hemodialysis (HD) patients and from 14.2% to 90.3% in peritoneal dialysis (PD) patients (Yasuda et al., 2002).

Compelling evidence has indicated that constipation is strongly associated with adverse clinical outcomes, including poor renal outcomes (Sumida et al., 2017), cardiovascular events (Park et al., 2025), and all-cause mortality (Halue et al., 2023). Accordingly, the prevalence of coronary heart disease and stroke in patients with constipation is 21% and 39% higher, respectively, and the all-cause mortality rate is 12% higher than that in patients without constipation (Sumida et al., 2019b). In addition, constipation contributes to physical discomfort, as well as reduced social functioning and quality of life (Bulbul et al., 2022). Thus, identifying risk factors for constipation holds important clinical significance in guiding clinical practice.

In addition to a sedentary lifestyle, low fiber and fluid intake, and concomitant medications (Shirazian and Radhakrishnan, 2010), the functional contraction of muscle groups, including the diaphragm and abdominal wall muscle, is essential to normal bowel movement. Consequently, the coordination of those muscle groups plays a crucial role in facilitating normal bowel function. Similarly, studies have confirmed that sarcopenia is closely associated with the severity of constipation in elderly individuals (Asaoka et al., 2021). This association may be attributed to a “gut-muscle axis”, wherein the gut microbiota and skeletal muscle exert bidirectional regulatory effects on one another. With advancing age, skeletal muscle undergoes degenerative changes, while the gut microbiota also exhibits distinct alterations, such as a reduction in microbial diversity, which may be linked to the quality and functional capacity of skeletal muscle (Zhang et al., 2020; Li et al., 2024). Unfortunately, progressive and systemic skeletal muscle disorder characterized by the accelerated loss of muscle mass and quality constitutes one of the prominent features of patients undergoing dialysis (Duarte et al., 2024). On this basis, we hypothesize that constipation in dialysis patients may be closely associated with the loss of skeletal muscle mass or quality.

Previously, accumulating evidence has indicated that pathological alterations in muscle mass or muscle quality are not only important predictive factors but also key pathogenic factors of various diseases. For instance, Sabatino et al. (2021) reported that in patients undergoing dialysis, sarcopenia is associated with an increased risk of adverse health outcomes, including functional decline, physical frailty-related falls, hospitalization, and even mortality. Thus, it is plausible that muscle dysfunction may serve as a predictive factor for the development of constipation.

Computed tomography (CT), which differentiates tissues on the basis of density, is widely recognized as the gold standard for assessing muscle quality across diverse populations. Some indicators, such as skeletal muscle mass and low-attenuation muscle, can be derived from CT data at the L1 or L3 vertebral level (Huang et al., 2024). Therefore, the present study aimed to assess the association between constipation and SMD in dialysis patients.

## Materials and methods

### Study population

**FIGURE 1:**
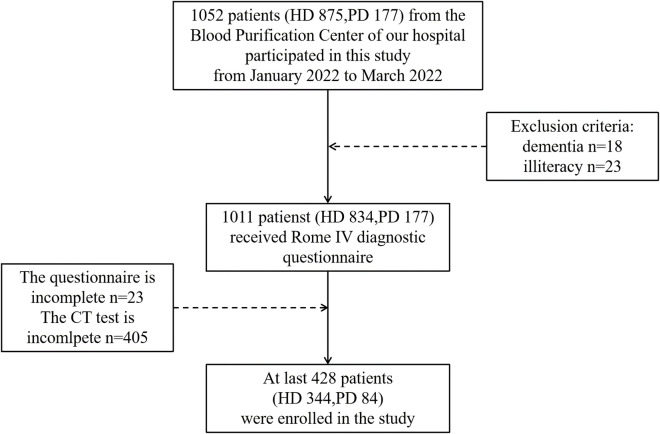
Flowchart of participants included in the study.

A total of 1052 patients (875 patients on HD; 177 patients on PD) were selected from three dialysis center registries and participated in this cross-sectional study from January 2022 to March 2022. The inclusion criteria were as follows: undergoing regular HD treatment (3 times per week) or PD treatment; an adult population (over 18 years old); and voluntary participation. The exclusion criteria included the following: irritable bowel syndrome, pathological constipation, a history of gastrointestinal surgery of less than 6 months, a diagnosis of gastroenteric tumors or colorectal inflammatory disease, regular dialysis treatment of less than 3 months, previous kidney transplantation, and cognitive deficits or illiteracy that would preclude the completion of a written questionnaire. Accordingly, patients with cognitive dysfunction (n = 18), those incapable of completing the questionnaire (n = 23), and those with no CT findings in the chest/abdomen or no imaging results at the L1 level (n = 405) were excluded. The Rome IV questionnaire and laboratory indicators were subsequently collected within 1 month. Finally, 428 patients (344 patients on HD; 84 patients on PD) who completed the constipation assessment and underwent CT scans were included in the study (the screening flow chart is presented in Figure 1).

Written informed consent was obtained from all individual participants. The study protocol was approved by the Ethics Committee for Clinical Research of Zhongda Hospital Affiliated with Southeast University (2021ZDSYLL139-P01, 24 June 2021).

### Assessment of constipation status

Validated questionnaires administered by 5 trained nurses were used to assess the participants’ constipation status. Every participant was asked the questions face-to-face on the questionnaires. Finally, the data were collected and managed.

Here, a Rome IV diagnostic questionnaire was downloaded from the Rome Foundation website (http://www.romecriteria.org/questionnaires/), and we conducted a questionnaire survey among patients on the day they underwent dialysis. The symptoms of functional constipation must include two or more of the following (Aziz et al., 2020): i Straining more than 25% of defecations. ii Lumpy or hard stools (Bristol Stool Form Scale type 1 or 2) accounted for more than 25% of defecations. iii Incomplete evacuation was associated with more than one-fourth (25%) of defecations. iv Anorectal obstruction/blockage was reported in more than one-fourth (25%) of the defecations. v Manual maneuvers to facilitate more than one-fourth (25%) of defecations. vi Fewer than three spontaneous bowel movements per week.

### Assessment of muscle quality

**FIGURE 2:**
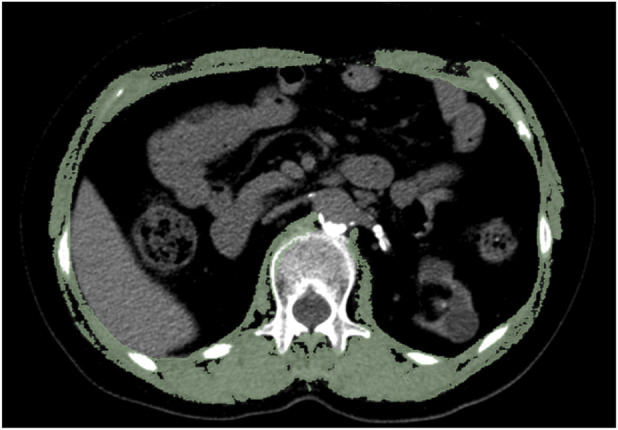
CT image showing skeletal muscle at the L1 level. The green section shows skeletal muscle at the L1 level, and the threshold ranges from −29 to +150 HU.

In the present study, CT scanners from General Electric (256-slice spiral CT scanners from General Electric Revolution CT) and Siemens (96-slice spiral CT scanners from SOMATOM Force CT) were used. One study revealed that L1 level-related indicators obtained from routine chest CT scans can serve as effective surrogate markers for evaluating overall skeletal muscle mass at the L3 level (Liu et al., 2023). Moreover, CT images at the L1 level are more easily obtained from opportunistic CT scans, without the need for patients to undergo additional examinations again. Therefore, we utilized a single axial CT scan at the L1 vertebra to analyze the skeletal muscle using 3D Slicer (version 5.0.3, https://www.slicer.org) (Fedorov et al., 2012). Accordingly, the tissue density was measured on CT images by the Hounsfield unit (HU) thresholds to identify different tissues; the CT analysis was blinded and was independently completed by 2 uniformly trained members. The CT values for the SMD range from −29 to +150 HU (Figure 2) (Golder et al., 2022). The skeletal muscle index (SMI) was derived by normalizing the muscle area relative to the patient’s height squared (cm^2^/m^2^), which served as an indicator of skeletal muscle mass. The SMD was assessed using the average radiation attenuation value across the entire muscle area at the L1 level, and a higher SMD on CT images reflects better quality and function of skeletal muscle.

### Statistical analysis

Continuous variables are expressed as the mean ± standard deviation if they followed a normal distribution or as the median (interquartile range, IQR) if they followed a skewed distribution. Categorical variables are presented as numbers (percentages). Continuous variables were compared using the Mann‒Whitney U test (for nonnormally distributed variables). The chi-square test was used to estimate the distributions of categorical variables. Univariate logistic regression analysis was used to identify risk factors for constipation, and the results are expressed as odds ratios (ORs) with 95% confidence intervals (CIs). Define the occurrence of constipation as a positive event, with an OR >1 as a protective factor and an OR <1 as a risk factor. A value of *P* < 0.05 (two-sided) indicated statistical significance. Afterward, we split patients into quartiles according to their SMD values at baseline, resulting in quartiles 1 (Q1), quartiles 2 (Q2), quartiles 3 (Q3), and quartiles 4 (Q4). Multivariable logistic regression models were constructed to assess ORs and 95% CIs for the association of SMD with the risk of incident constipation, with the lowest quartile used as the reference. Factors that were statistically significant by univariate analysis in the association of constipation were adjusted in the multivariate logistic regression models. Before multivariate logistic regression, the collinearity between independent variables was analyzed by detecting the tolerance and variance inflation factor. Data analysis was performed using IBM SPSS Statistics for Mac, version 25. To analyze the robustness of the primary results, receiver operating characteristic (ROC) curves and the area under the curve (AUC) were constructed. Using the ‘sample’ function in R (R for Mac Version 4.1.2), the data were divided into a training set and a test set in a 7:3 distribution.

## Results

### Patient characteristics and clinical features

Overall, 428 patients were included. The mean age was 56.09 (±15.17) years. The percentage of male participants was 42.99%. The mean dialysis vintage was 7.31 years (1–32 years). In accordance with the Rome IV diagnostic criteria, the prevalence of constipation among dialysis patients was 62.62%. Table 1 shows the descriptive clinical features of dialysis patients with and without constipation.

**TABLE 1:** Baseline characteristics.

Variables	Total (n = 428)	No constipation (n = 160)	Constipation (n = 268)	*P*
Age (years)	56.09 ± 15.17	50.97 ± 14.52	59.14 ± 14.74	<0.001^a^
Sex, n (%)				0.076
Male	184 (42.99)	60 (37.50)	124 (46.27)	
Female	244 (57.01)	100 (62.50)	144 (53.73)	
BMI (kg/m^2^)	22.84 ± 3.77	22.15 ± 3.37	23.25 ± 3.94	0.003^a^
Dialysis vintage (years)	7.32 ± 5.83	8.07 ± 6.09	6.86 ± 5.63	0.037^a^
Dialysis type, n (%)				0.859
Hemodialysis	327 (76.40)	123 (76.88)	204 (76.12)	
Peritoneal dialysis	101 (23.60)	37 (23.12)	64 (23.88)	
Primary disease				
Hypertension, n (%)	253 (59.11)	94 (58.75)	159 (59.33)	0.906
Diabetes, n (%)	80 (18.69)	21 (13.12)	59 (22.01)	0.022^a^
Medication history				
Cathartic, n (%)	13 (3.04)	2 (1.25)	11 (4.10)	0.170
Antihypertensive drug, n (%)	218 (50.93)	76 (47.50)	142 (52.99)	0.272
Phosphorus binder, n (%)	177 (41.36)	64 (40.00)	113 (42.16)	0.660
Laboratory results				
Hemoglobin (g/L)	104.40 ± 19.35	107.60 ± 20.00	102.49 ± 18.73	0.009^a^
Serum albumin (g/L)	37.48 ± 4.99	38.15 ± 5.15	37.08 ± 4.86	0.034^a^
Serum calcium (mmol/L)	2.21 ± 0.27	2.20 ± 0.28	2.21 ± 0.26	0.752
Serum phosphate (g/L)	1.70 ± 0.60	1.80 ± 0.57	1.64 ± 0.62	0.010^a^
Serum PTH (pg/mL)	372.23 ± 386.64	369.26 ± 394.04	374.00 ± 382.92	0.906

BMI, body mass index; PTH, parathyroid hormone. The values are presented as the mean ± SD.

^a^ indicates statistical significance (p < 0.05).

## SMD

**FIGURE 3:**
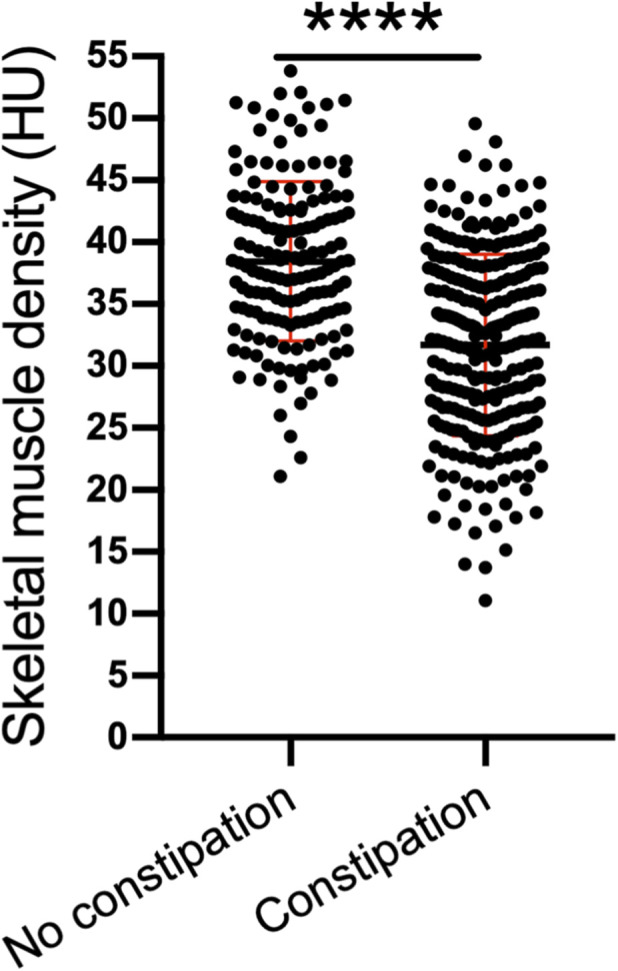
Skeletal muscle density in patients. ****P < 0.0001.

We found that compared with that of patients with no constipation, the SMD of patients with constipation was significantly lower (31.70 ± 7.33 HU vs. 38.44 ± 6.44 HU; P < 0.001) (Figure 3).

### Risk factors for constipation in dialysis patients

The risk factors for constipation in dialysis patients were subsequently explored. In our study, univariate logistic regression analysis revealed that older age, a history of diabetes, lower serum albumin levels, lower hemoglobin levels, lower phosphorus levels and lower SMDs were risk factors for constipation (*P* < 0.05), whereas lower BMI and longer dialysis vintage were protective factors against constipation (all *P* < 0.05) (Table 2).

**TABLE 2:** Univariate logistic regression analyses for constipation in all dialysis patients.

Variables	β	S.E	Z	*P*	Or (95%CI)
Age (years)	0.04	0.01	5.25	<0.001^a^	1.04 (1.02∼1.05)
Sex					
Male					1.00 (reference)
Female	−0.36	0.20	−1.77	0.077	0.70 (0.47∼1.04)
BMI (kg/m^2^)	0.08	0.03	2.89	0.004^a^	1.09 (1.03∼1.15)
Dialysis vintage (years)	−0.04	0.02	−2.07	0.039^a^	0.97 (0.93∼0.99)
Dialysis type					
Hemodialysis					1.00 (reference)
Peritoneal dialysis	0.04	0.24	0.18	0.859	1.04 (0.66∼1.66)
Hypertension	0.02	0.20	0.12	0.906	1.02 (0.69∼1.53)
Diabetes	0.63	0.28	2.26	0.024^a^	1.87 (1.09∼3.21)
Cathartic	1.22	0.78	1.57	0.116	3.38 (0.74∼15.45)
Antihypertensive drug	0.22	0.20	1.10	0.272	1.25 (0.84∼1.84)
Phosphorus binder	0.09	0.20	0.44	0.660	1.09 (0.73∼1.63)
Hemoglobin (g/L)	−0.01	0.01	−2.57	0.010^a^	0.99 (0.98∼0.99)
Albumin (g/L)	−0.04	0.02	−2.11	0.035^a^	0.96 (0.92∼0.99)
Calcium (mmol/L)	0.12	0.39	0.32	0.752	1.13 (0.53∼2.41)
Serum phosphate, (mmol/L)	−0.44	0.17	−2.56	0.011^a^	0.64 (0.46∼0.90)
PTH (pg/mL)	0.00	0.00	0.12	0.906	1.00 (1.00∼1.00)
SMD (HU)	−0.14	0.02	−7.97	<0.001^a^	0.87 (0.84∼0.90)
SMI (cm^2^/m^2^)	−0.39	0.05	−7.87	<0.001^a^	0.68 (0.61∼0.75)

BMI, body mass index; SMI, skeletal muscle index; SMD, skeletal muscle density; PTH, parathyroid hormone. The values are presented as the mean ± SD.

^a^ indicates statistical significance (p < 0.05).

The multivariate-adjusted ORs and 95% CIs for incident constipation according to the continuous values and quartiles of the SMD are summarized in Table 3. When regarded as a continuous variable, the SMD (OR 0.87, 95% CI 0.84–0.90; p < 0.001) was independently correlated with the occurrence of constipation. Dialysis patients were further grouped by quartiles of SMD. Compared with those in the lowest quartile, individuals in the highest quartile of the SMD were 0.21-fold more likely to have incident constipation after adjusting for age, dialysis vintage, hemoglobin level, serum phosphate level and history of diabetes, SMI and BMI.

**TABLE 3:** Logistic regression analyses for constipation according to SMD.

Variables	Model 1		Model 2		Model 3	
	Or (95%CI)	*P*	Or (95%CI)	*P*	Or (95%CI)	*P*
Continuous variable						
SMD	0.87 (0.84∼0.90)	<0.001^a^	0.87 (0.83∼0.91)	<0.001^a^	0.90 (0.84∼0.98)	0.009^a^
Categorical variable						
Q 1	1.00 (reference)		1.00 (reference)		1.00 (reference)	
Q 2	0.13 (0.05∼0.31)	<0.001^a^	0.15 (0.06∼0.36)	<0.001^a^	0.26 (0.10∼0.70)	0.008^a^
Q 3	0.08 (0.03∼0.19)	<0.001^a^	0.10 (0.04∼0.24)	<0.001^a^	0.25 (0.08∼0.80)	0.019^a^
Q 4	0.05 (0.02∼0.11)	<0.001^a^	0.05 (0.02∼0.14)	<0.001^a^	0.21 (0.05∼0.80)	0.022^a^

The Q1 (quartile 1) group of the SMD, was considered a reference group. Model 1, unadjusted model. Model 2 was adjusted for age, dialysis vintage, hemoglobin, albumin, serum phosphate and history of diabetes. Model 3, adjusted for “above + SMI + BMI”.

SMD, skeletal muscle density; SMI, skeletal muscle index; BMI, body mass index. The values are presented as the mean ± SD.

^a^ indicates statistical significance (p < 0.05).

### Nomogram construction and validation

**FIGURE 4:**
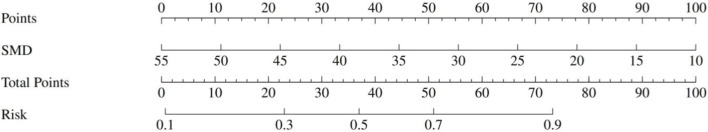
Nomogram of the risk of constipation.

**FIGURE 5:**
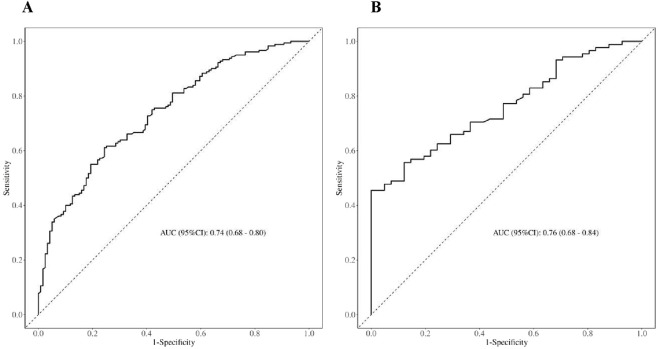
ROC curves of the nomogram. The parameter that was analyzed in this regression was the SMD. **(A)** is training; **(B)** is validation.

Factors that were significant in multivariable regression were used to develop a nomogram to assess the risk of constipation in dialysis patients (Figure 4). To determine the accuracy of the models, we plotted the ROC curve of the nomogram in the training set and test set (Figure 5). The results suggested that the AUC of the model in the training data was 0.74, with 39% sensitivity and 24% specificity. The AUC of the model for the test data was 0.76, with 35% sensitivity and 29% specificity.

## Discussion

In this study, the potential association between constipation and SMD in patients on maintenance dialysis was explored. We found that the prevalence of constipation among dialysis patients was 62.62%. Furthermore, for the first time, we demonstrated that a low SMD is a risk factor for constipation and that this association is independent of sex.

Constipation is a common, burdensome and, in some cases, debilitating problem for patients with end-stage renal disease. Accordingly, constipation may increase the risk of hyperkalemia in dialysis patients (Kalantar-Zadeh et al., 2020). Robust evidence has indicated that HD patients excrete 3 times more potassium in feces than the general population does; consequently, the abundance of potassium in the stool may increase intestinal potassium absorption (Sumida et al., 2021). Second, inaccurate dry weight assessment attributable to constipation may lead to hypovolaemic or hypervolemic states, along with their associated complications. More importantly, constipation is linked to an increased risk of adverse clinical events, including coronary heart disease, stroke, and all-cause mortality. Despite these adverse effects, constipation remains underrecognized by clinicians and underreported by patients in clinical settings (Moschen et al., 2022; Jain, 2023). Thus, clarifying the prevalence and risk factors for constipation in this population is highly important. In the present study, we reported that the prevalence of constipation among the included dialysis patients was 62.62%. The high prevalence of constipation thus warrants widespread attention in clinical practice.

Extensive evidence has shown that constipation is associated with older age, female sex, decreased physical activity, concomitant medication, and stressful life events (Cui et al., 2024; Kilgore and Khlevner, 2024). However, the pathogenesis of constipation remains unclear. It is well known that the physiological defecation process is a product of the coordinated contraction and relaxation of the abdominal muscles, rectum, anal sphincter, and pelvic floor muscles. During defecation, the relaxation of the internal and external sphincters and puborectalis muscle facilitates fecal excretion (Rao, 2004), a phenomenon that collectively indicates that muscle function plays a crucial role in the development of constipation. In clinical practice, early identification of constipation in this patient population remains challenging. In the present study, we demonstrated for the first time that a low SMD is an independent risk factor for constipation among patients on maintenance dialysis. Thus, our findings are biologically plausible. Accordingly, improving the low SMD may represent an effective strategy for alleviating constipation in this group.

Data from a single CT slice are sufficient to characterize the status of whole-body muscles, fat, and other components. The objective and stability of its data make it the gold standard for noninvasive assessment of body composition (Beaudart et al., 2016). Among its various applications, the evaluation of sarcopenia using L3-level CT data has been widely adopted in clinical trials focused on oncology and other fields (Kays et al., 2020; Mangus et al., 2017; Oh and Lee, 2020; Springer et al., 2017; Tabourin et al., 2022; Xiao et al., 2020; Yip et al., 2014). However, among the dialysis population, clinical practice frequently involves chest CT examinations, largely because of the high prevalence of conditions such as pulmonary infection and heart failure. In contrast, abdominal CT is performed far less frequently in clinical practice, resulting in the limited availability of L3-level images. Notably, against the global backdrop of the COVID-19 pandemic, all hospitalized patients routinely undergo chest CT imaging, and this widespread availability has markedly enhanced the clinical utility of L1-level image analysis while eliminating additional economic burden. Therefore, in recent years, a growing number of scholars have turned their attention to the use of opportunistic CT to evaluate muscle mass or muscle quantify. Multiple studies (Derstine et al., 2018; Recio-Boiles et al., 2018) have shown that L1-level CT images serve as an excellent alternative to L3-level images and are representative for body composition analysis. Therefore, in this study, the selection of CT images to investigate the association between body composition indicators derived from these images and constipation in dialysis patients has significant clinical practical value.

Previously, increasing evidence has indicated that age and sex have nonnegligible effects on skeletal muscle mass and quality (Ichinose et al., 2024). However, in our study, we found that a low SMD is a risk factor for constipation, with this association being independent of age and sex. While these findings may seem to contradict those of prior studies (Wu et al., 2016; Gharpure et al., 2023), the study population itself may be a key factor explaining this discrepancy. Notably, persistent muscle protein degradation and loss are hallmark features of dialysis patients (Troutman et al., 2024). Existing studies have consistently indicated that sarcopenia is highly prevalent among dialysis-dependent patients with CKD. The pathogenesis of sarcopenia in this specific population can be attributed primarily to two interconnected mechanisms. First, metabolic disturbances and chronic inflammatory responses induced by renal failure act as core drivers of the development and progression of sarcopenia. Specifically, the aforementioned metabolic disturbances encompass a spectrum of pathological conditions, including malnutrition (arising from impaired nutrient absorption and utilization), insulin resistance, diabetic nephropathy, acid‒base disturbances, and electrolyte disorders (Fahal, 2014). With respect to inflammatory processes, the persistent release of proinflammatory cytokines (e.g., tumor necrosis factor-α and interleukin-6) and oxidative stress-induced cellular damage represent the core pathological drivers of muscle wasting (Stenvinkel and Alvestrand, 2002). Second, dialysis procedures themselves have a notable effect on muscle metabolism. Clinical evidence has demonstrated that dialysis can trigger the activation of skeletal muscle protein degradation pathways while concurrently inhibiting protein synthesis. Notably, such metabolic perturbations tend to persist into the postdialysis period, extending beyond the duration of the dialysis session, resulting in a persistent negative protein balance and subsequent progressive loss of muscle mass (Rajakaruna et al., 2015). Thus, our findings may provide novel insight into the increased prevalence of constipation among dialysis patients.

Although the specificity and sensitivity of the SMD for predicting constipation in dialysis patients were suboptimal in the present study, the pathogenesis of constipation is inherently complex and modulated by a multitude of factors, which precludes a comprehensive exploration of the determinants of constipation from a single dimension. Moreover, clinical interventions for constipation remain limited in both scope and efficacy (Hojo et al., 2025). Therefore, the findings of our study still provide a new avenue for developing constipation treatment strategies—specifically, improving SMD may help alleviate constipation-related symptoms in dialysis patients. Approximately one-third of patients with constipation experience evacuation disorders, and dyssynergic defecation is a common cause of pelvic evacuation disorders. In cases of dyssynergic defecation, the coordination between abdominal and pelvic floor muscles is impaired during defecation, preventing patients from achieving normal bowel movement (Sadeghi et al., 2023). In contrast, L1-level CT images characterize primarily the status of the psoas major muscles and abdominal wall muscles. These muscles play a notable role in generating intra-abdominal pressure to facilitate defecation. This finding thus provides a plausible explanation for the finding that low SMD is a significant factor influencing constipation. Notably, Gao et al. (2019) reported that exercise, as a feasible and effective intervention, can reduce the occurrence of constipation. Similarly, Cui et al. (2024) reported that physical activity constitutes an effective approach for alleviating symptoms of constipation. Several potential mechanisms may underlie the beneficial effects of physical activity on constipation. First, physical activity can effectively enhance intestinal motility, and the impairment or decrease in intestinal motility is a well-recognized key contributing factor to constipation (Liu et al., 1993). Specifically, in individuals with chronic constipation symptoms who also present with low physical activity levels, regular engagement in structured physical activity has been shown to significantly shorten the transit time of the cecum and colon (De Schryver et al., 2005). Additionally, accumulating evidence indicates that specific modalities of physical activity, such as aerobic exercise and core-strengthening exercise, can further reduce colonic transit time, contributing to the alleviation of constipation symptoms (Song et al., 2021; Kim et al., 2014). Therefore, it is reasonable to propose that increasing muscle density may reduce the prevalence of constipation to some extent, although further well-designed clinical studies are needed.

This study has several limitations. First, it adopted a single-center, cross-sectional, correlation study. Owing to this design, the exact mechanism of low SMD in patients with constipation has not been elucidated, nor can it explain the causal relationship between constipation and low SMD. Moreover, since we collected data from the L1 level in patients’ opportunistic CT scans, we were unable to synchronize the acquisition of CT data with the assessment of constipation. However, all the patients included in this study were dialysis patients in a stable disease stage, and their SMD and SMI data are unlikely to significantly change in the short term. Second, the study enrolled patients who were receiving dialysis; the relationship among patients with CKD who are not receiving dialysis needs evaluation. Third, for some older patients, scoring needs to be performed with staff assistance; therefore, there may be certain deviations in the data. Finally, owing to fluid restriction and reduced physical activity in dialysis patients, we did not collect the above indicators when we analyzed the risk factors for constipation. Furthermore, analyses of patients’ physical activity and dietary fiber intake were not included in this study, which we recognize as limitations. Although controversy remains regarding the impact of exercise and dietary fiber intake on constipation, the findings of this study—namely, that SMD is a risk factor for constipation in dialysis patients—may serve as a new target. In the future, we aim to adopt an RCT method to explore the effect of increasing the SMD on improving constipation in HD patients.

In conclusion, patients on dialysis have a higher prevalence of constipation. A low SMD is an independent risk factor for constipation. Our findings provide a new perspective on the causes of susceptibility to constipation among dialysis patients.

## Data Availability

The raw data supporting the conclusions of this article will be made available by the authors, without undue reservation.
